# Moyamoya disease and pregnancy: case reports and criteria for successful vaginal delivery

**DOI:** 10.1002/ccr3.199

**Published:** 2015-02-04

**Authors:** Kiguna Sei, Hidenori Sasa, Kenichi Furuya

**Affiliations:** Department of Obstetrics and Gynecology, National Defense Medical College3-2 Namiki, Tokorozawa, Saitama, Japan

**Keywords:** Cesarean delivery, extradural anesthesia, general anesthesia, Moyamoya disease, pregnancy, vaginal delivery

## Abstract

Based on our experience with seven deliveries (five cesarean and two vaginal deliveries) in five women with Moyamoya disease, we discussed the appropriate method of delivery and anesthesia for patients with Moyamoya disease. In certain conditions, women with Moyamoya disease can successfully undergo vaginal delivery.

## Introduction

Moyamoya disease (MMD) is a rare cerebrovascular disease characterized by progressive stenosis or occlusion of the bilateral internal carotid arteries and anterior or median cerebral arteries. The disease may initially present with ischemic (loss of consciousness, seizures, headaches, and infarction) or hemorrhagic symptoms. Pediatric patients present with typical ischemic symptoms in their first attacks, whereas adult patients present with hemorrhagic symptoms 1. The prevalence of MMD is the highest in Japan, with an incidence of 3.14 cases per 100,000 individuals, nearly 10-fold higher than that in European populations. The female-to-male ratio is approximately 2:1 2.

Currently, there is no treatment to reverse the primary disease process, and stroke prevention is therefore the main aim of treatment. Surgical revascularization is performed to improve cerebral blood flow in both children and adults. However, the efficacy of this approach and long-term outcomes, particular in patients with hemorrhagic symptoms, remain unclear.

**Table 1:** Case characteristics

Case No.	Age	Parity	Type of initial attack	Presence of surgical revascularization (Y/N)	Gestational week	Delivery/anesthetic methods
1	28	0	Ischemic	Y	33	Cesarean General
2	28	0	Hemorrhagic	Y	37	Cesarean CSEA
	32	1			37	Cesarean CSEA
3	36	1	Ischemic	N	37	Cesarean General
	38	2			37	Cesarean General
4	30	0	Ischemic	Y	38	Vaginal Epidural
5	24	0	Ischemic	Y	38	Vaginal Epidural

CSEA, combined spinal epidural anesthesia.

There are no consistent guidelines for managing pregnancy and delivery in women with MMD, and more than 70% of pregnant women with MMD undergo cesarean delivery to avoid the risk of intracerebral hemorrhage or cerebral infarction 3.

In our experience with five MMD patients (Table1), there were two cases of successful vaginal delivery using epidural anesthesia and five cases of cesarean delivery (three with general anesthesia, two cases with subarachnoid and epidural analgesia). We examined the medical records of these patients, and discussed the appropriate methods of delivery for pregnant women with MMD.

## Case Reports

### Case 1

A 28-year-old nulliparous woman initially presented with an ischemic attack (right hemiparesis) at the age of 20 years. She had a family history of MMD on the paternal side, and we therefore made a diagnosis of familial MMD. Although she underwent a bypass surgery immediately after diagnosis and magnetic resonance angiography revealed good blood circulation, she experienced several ischemic attacks after the operation and throughout pregnancy as well. She was diagnosed with pregnancy-induced hypertension (PIH) at 33 weeks of gestation, and developed HELLP syndrome (hemolysis, elevated liver enzymes, and low platelets) at 35 weeks. An emergency cesarean delivery under general anesthesia was performed because of thrombocytopenia caused by HELLP syndrome. The male neonate had a birth weight of 1692 g and Apgar scores of 9 and 10 at 1 and 5 min after birth, respectively.

### Case 2

A 28-year-old nulliparous woman experienced intracerebral hemorrhage at the age of 19 years. Cerebral revascularization and hematoma evacuation were then performed immediately. Thereafter, she was asymptomatic, but lesions indicative of infarction were detected by magnetic resonance imaging ever since.

Her blood pressure began to increase during the first trimester, and she was diagnosed with PIH at 30 weeks of gestation; pharmaceutical treatment was then initiated. In order to stabilize her blood pressure and avoid recurrence of hemorrhage and infarction, she underwent a cesarean delivery under general anesthesia at 37 weeks of gestation. The male neonate had a birth weight of 3030 g and Apgar scores of 9 and 10 at 1 and 5 min after birth, respectively.

Four years later, the patient conceived again and showed no signs of hypertension. She underwent a cesarean delivery with subarachnoid analgesia and epidural analgesia. The male neonate had a birth weight of 2540 g and Apgar scores of 9 and 10 at 1 and 5 min after birth, respectively.

### Case 3

A 36-year-old woman (para 1) did not inform her doctor that she had MMD during her first pregnancy, and therefore underwent a normal vaginal delivery. No medical data concerning the presence or absence of MMD during this pregnancy were obtained.

She first experienced an ischemic attack that involved hypocapnia-induced fainting at the age of 5 years. No surgical revascularization was performed, and she experienced frequent attacks thereafter that involved fainting and headaches.

Her second pregnancy was detected during hospitalization for treatment of medical poisoning due to an overdose of antidepressants. She often complained of anxiety and frequent hypocapnia and headaches during pregnancy. Her blood pressure began to increase at 32 weeks of gestation, and pharmaceutical treatment was then initiated. She underwent an elective cesarean delivery at 37 weeks of gestation. Her psychiatric condition and safe control of ventilation and blood pressure were the important factors for selecting general anesthesia. The male neonate had a birth weight of 2699 g and Apgar scores of 6 at 1 and 5 min after birth.

Two years after the second pregnancy, the patient conceived again. Ischemic attacks related to MMD were observed during this third pregnancy. She underwent a cesarean section under general anesthesia at 37 weeks of gestation and delivered a female neonate with a birth weight of 2750 g and Apgar scores of 5 and 6 at 1 and 5 min after birth, respectively.

### Case 4

A 30-year-old nulliparous woman presented with fainting due to hypocapnia associated with MMD at the age of 7 years. Surgical revascularization was performed at 9 years of age. She experienced no symptoms during pregnancy. At 38 weeks of gestation, she underwent vaginal delivery with epidural anesthesia; labor proceeded uneventfully, with no appearance of symptoms. To prevent intracranial pressure increase due to bearing down, vacuum extraction was performed. She delivered a female neonate with a birth weight of 2545 g and Apgar scores of 9 and 10 at 1 and 5 min after birth, respectively.

### Case 5

A 24-year-old nulliparous woman initially presented with fainting and seizures at 6 years of age, which were diagnosed as MMD-associated ischemic attacks, and surgical revascularization was performed immediately. She was asymptomatic thereafter, including during her pregnancy. She underwent vaginal delivery at 38 weeks of gestation and delivered successfully by vacuum extraction under epidural anesthesia. The male neonate had a birth weight of 3120 g and Apgar scores 9 at 1 and 5 min after birth, respectively.

## Discussion

The most important aspect in our management approach during pregnancy in our cases of MMD was the prevention of critical attacks, which cause maternal death or severe neurological symptoms including intracerebral hemorrhage and infarction. All seven deliveries were successful, with no MMD-related attacks or symptoms during the peripartum period.

Although there is no evidence that women with MMD have an increased risk of ischemic or hemorrhagic attacks during pregnancy, pregnancy itself is recognized as one of the risk factors for stroke (intracranial hemorrhage and infarction). Treadwell et al. reported a three-fold increase in the incidence of stroke in pregnant women compared with that in nonpregnant women. In pregnant women, the mortality rates attributed to intracranial hemorrhage and infarction range from 8 to 15% 4. PIH is recognized as a risk factor for intracranial hemorrhage, and Sharshar et al. showed that 44% of intraparenchymal hemorrhage in pregnant women presents as eclampsia 5. Hormonal changes during pregnancy increase blood volume by 40–50% and affect the systemic arteries, and may lead to fragmentation of reticulin fibers, resulting in the loss of normal corrugation of elastic fibers 6. In addition, hypercoagulability, venous stasis, and vascular endothelial injury during pregnancy are regarded as contributing factors to intracranial infarction 7. Therefore, pregnant women with MMD who have vulnerable cerebral vessels may have a higher risk of stroke than pregnant women without MMD.

We recognized that blood pressure control is most critical for pregnant women with MMD. The vessels of pregnant women are vulnerable, and rapid increases in blood pressure can cause intracranial hemorrhage. Conversely, rapid decreases in blood pressure can also cause Moyamoya vessel spasms that lead to ischemic attacks, including infarction. These rapid changes in blood pressure are observed most frequently during labor, and also in patients with PIH. It is important to determine the method of delivery based on strict assessments of each case of MMD.

More than 70% of pregnant women with MMD undergo cesarean delivery 3. Cesarean delivery is the method of choice to avoid intracranial hemorrhage due to hypertension during labor, although sudden intraoperative hypotension caused by induction of analgesia and postoperative hypercoagulation, which is more severe than that caused by vaginal delivery, can cause infarction 8. Therefore, we considered that cesarean delivery was more adequate for the patients at higher risk of intracranial hemorrhage than ischemic attacks, such as those who had ever experienced a hemorrhagic attack, patients with PIH during pregnancy, and patients who had not undergone surgical revascularization.

Miyamoto et al. 9 demonstrated that surgical revascularization, a commonly used MMD treatment, decreased not only the incidence of ischemic attack but also the incidence of hemorrhage in patients whose initial attack involved ischemic symptoms. Hemorrhage, as the initial attack, is considered more severe than ischemic symptoms, and the role of surgical revascularization in the prevention of re-hemorrhage is unclear.

When considering cesarean delivery, we also recognized the importance of determining the appropriate anesthetic method. Local analgesia, including subarachnoid or epidural analgesia, is commonly used for cesarean delivery. However, these two methods induce sudden hypotension, which can cause ischemic attacks in MMD patients. In general anesthesia, temporary hypertension due to pressor responses during trachial intubation can cause intracranial hemorrhage. Stoelting et al. 10 showed that a short-duration direct laryngoscopy combined with laryngotracheal lidocaine administration minimized pressor responses. Therefore, general anesthesia with gentle and direct-vision intubation can control blood pressure better than regional anesthesia. Control of ventilation is also necessary for the prevention of hypocapnia, which can lead to seizures and fainting, and complicate delivery. General anesthesia may also be appropriate for patients with severe anxiety, which results in a higher risk of hypocapnia. Neonate unconsciousness caused by the transition of anesthetic drugs to the neonate is another problem in cesarean delivery with general anesthesia, and the cooperation of neonatologists is desirable. We experienced four cesarean deliveries under general anesthesia, and observed relatively low Apgar scores in two neonates with the same mother (case 3). The low Apgar scores were considered the result of the transition of multiple antidepressants to the neonate. The other two neonates with general anesthesia needed no special care.

We recommended vaginal delivery to patients who had a lower risk of intracranial hemorrhage, such as those who underwent surgical revascularization without a history of hemorrhage or PIH. Two vaginal deliveries were successfully performed using epidural anesthesia. Successful epidural anesthesia can decrease endogenous catecholamine levels with the onset of labor pain relief 11, leading to a reduction in systemic vascular resistance. In addition, relief of labor pain can prevent anxiety-induced hypocapnia. Vacuum extraction was performed to avoid temporary hypertension caused by bearing down before delivery. Extraction matching uterine contractions without bearing down stabilized blood pressure, and no strokes occurred in either patient.

Recently, it has been reported that cesarean delivery is associated with higher risks of severe maternal morbidity than vaginal delivery 12. Therefore, safe prevention of primary cesarean delivery is necessary, including among women with MMD. Vaginal delivery may be considered in women with MMD at a lower risk of intracranial hemorrhage because of their type of primary attack and history of surgical revascularization. In addition, appropriate anesthesia methods for cesarean delivery should be considered.
